# Supplementary material for: The Molecular Evolution, Structure, and Function of Coproporphyrinogen Oxidase and Protoporphyrinogen Oxidase in Prokaryotes
Source: Biology (Basel). 2023 Dec 15;12(12):1527. doi: 10.3390/biology12121527 (PMC10740692; doi:10.3390/biology12121527)
Supplement: Supplementary file 1 [file biology-12-01527-s001.zip › biology-2692858-supplementary.pdf]

# The Molecular Evolution, Structure, and Function of Coproporphyrinogen Oxidase and Protoporphyrinogen Oxidase in Prokaryotes

**Marcel Zámocký [1,2], Stefan Hofbauer [3], Thomas Gabler [3] and Paul G. Furtmüller [3,*]**

[1] Laboratory of Phylogenomic Ecology, Institute of Molecular Biology, Slovak Academy of Sciences, Dúbravská Cesta 21, SK-84551 Bratislava, Slovakia; marcel.zamocky@savba.sk
[2] Department of Inorganic Chemistry, Faculty of Natural Sciences, Comenius University in Bratislava, Mlynská Dolina, Ilkovičova 6, SK-84215 Bratislava, Slovakia
[3] Institute of Biochemistry, Department of Chemistry, University of Natural Resources and Life Sciences, Vienna, Muthgasse 18, A-1190 Vienna, Austria; stefan.hofbauer@boku.ac.at (S.H.);
thomas.gabler@boku.ac.at (T.G.)
* Correspondence: paul.furtmueller@boku.ac.at; Tel.: +43-147654-77277

**Table S1: 74 selected full-length CgoX and PgoX protein sequences**

All sequences beginning with "WP" were obtained from the NCBI database, while all other sequences are from the Uniprot database.

>**WP_124059669.1**_Bacillus_subtilis_Bacillota

MSDGKKHVVIIGGGITGLAAAFYMEKEIKEKNLPLELTLVEASPRVGGKIQTVKKDGYIIERGPDSFLER

KKSAPQLVKDLGLKHLLVNNATGQSYVLVNRTLHPMPKGAVMGIPTKIAPFVSTGLFSLSGKARAAMDFI

LPASKTKDDQSLGEFFRRRVGDEVVENLIEPLLSGIYAGDIDKLSLMSTFPQFYQTEQKHRSLILGMKKT

RPQGSGQQLTAKKQGQFQTLSTGLQTLVEEIEKQLKLTKVYKGTKVTKLSRGGSGYSLELDNGVTLDADS

VIVTAPHKAAVGMLSELPAISHLKNMHSTSVANVALGFPEGSVQMEHEGTGFVISRNSDFAITACTWTNK

KWPHAAPEGKTLLRAYVGKAGDESIVDLSDNDIINIVLEDLKKVMNINGEPEMTCVTRWHESMPQYHVGH

KQRIKELREALASAYPGVYMTGASFEGVGIPDCIDQGKAAVSDALTYLFS

>**WP_046336255.1**_Listeria_monocytogenes_Bacillota

MKHIVIIGGGLSGLAAAYELQKTHPNYTWELVEKDEKLGGKFETVKRDGFLIEKGPDSFLARKPAGVGLV

KDLGLEDKLIANATGRSYIYHQKALHPIPEGSVMGIPTDKEALLASTLVSEIGKARALQEPTMERNNQER

DQALGDFFEARFGKELVKTIIEPLLSGIYAGDIYKMSLRATFPQFEQTVKKYGNLMDGLKESSMQTTGTK

ATIGAFRTLEGGLDTLPKAIAAALPKENLHTAKQATQIVKKNNAYEISFADGDKMEADGVIIAATHDALI

HLLAETTTEPFAGQPLTTLATVSLAYNEQDVPILPDGTGYLVARTAPYKTTACTWVQKKWPHMVPKNKML

LRGFVGKAGETWLEQASDEAIVSAVLRDYAEIMDLHAAPLFYEVSRMKSAMPQYLVNHQDRLKQLKKNIK
TDYPGVFFAGMSYEGVGIPDCIAGAQTAAKELVDYLEEV

>**WP_185426979.1**_Listeria_weihenstephanensis_Bacillota
MKKIVIIGGGLTGLSAAHYLNKQVDGEAVDWQLFEADDHFGGKFNTVHRDGFIIEKGPDSFLARKPAGMA
LVEELGLTDQLITNATGKSYIYHEKALHPIPEGSVMGIPTDIRALLESDLLTEDGKIRALEELMLESNVT
DADQSIGDFFEYRFGKEMVVHLIEPLLAGIYAGNIYEMSLRATFPQFEQTAKEHRSLMYGLKSHRPATVN
TTGTAKTVGAFRTLDGGLSTLTDALVASLPAERLHACTKVKSIESTTVVLESGERIVADAIIIAATHDVV
LSLLDVPALQPLKAQPMTSLATISLAFDKEAVPEIPEGTGFLVARTGDYHITACTWVHEKWPHMVPDGKI
LLRGFLGKAGQVGLLDQTDQEMADKVQADLKEMIGLRGKPLFYEVSRMKEAMPQYTVGHQERLKKVEADL
RESHPTVFLAGMSYRGVGIPDCIDGGKEAANEALALLEQVRS

>**WP_119905274.1**_Brochothrix_thermosphacta_Bacillota
MKKVAIIGGGLSGLTAAFYLARQNKNIDWQVYEASARFGGHIKTVHKQGYIIEKGPDSFLARKPAGMQLI
HDLKLDSELVKNATGQSYIYQNKIFHPIPEGSIMGIPTKLKPFLKSKLFSPLGKVRVLYELIKPADRSKE
DQSLGDFFEKRFGKEMVTHLIEPLFSGIYAGNLYQMSLRATYPAMETLLNENGSILKGLRKQRVQTTGTK
QTIGAFRTLKGGMTQLVDELVKQLPTERCHLNKAIENIEKKEDRYHLLFKNGEIEVVDEIIFGINHLRAL
ELMPQYESPLKNQKIASSATLSFGFKIDDVPSLPEGTGFLMTREKDEHMTACTWVHTKWPHMVPENRVLL
RVFLGRSNLSNVTELADDELISLARTYLQDIMGVTAEPEVTEISKMNMAMPQYDIGHTERVASLLEKTRH
QREGLHFIGMSYEGVGIPDCIQLAKETVKNM

>**WP_120116355.1**_Siminovitchia_terrae_Bacillota
MAEDKKKVVVIGGGITGLSAAYYLQKTIHEKKLPCDVLLVEASHKLGGKFQTVYKDGFTIERGPDSFLER
KKSAAELAIDVGLKDELVNNTAGRSFVLANEKLHPMPKGAVMGIPTKITPFITTDLFSIQGKMRAAADFV
MPRSKVSGDQSLGGFFRRRLGDEVVENLVEPLLSGIYAGDIDRLSLMSTFPQFYQVEQKYRSLIMGMKKT
TPSGPKKEVSAKNQGIFLTLKGGLQSLVKAIEEKLEPSSILKGTRVVSIVNNNEKGYKLQLNNGEWLEAD
SIILTLPHFALADLFPDNPVIQGMKEMPATSVATVAMAFDAKDVDDHLDGTGFVVSRNSDYTITACTWTH
KKWPHTTPEGKVLLRCYVGRAGEETVVDLSDEEIERIVLADLKRTMDLSAKPNFTIVTRWKQAMPQYTVG
HKERIDKIKSDFQNNMPGVFIAGSSYAGLGIPDCIDQGKAAVDDVLRHIH

>**WP_237419869.1**_Geobacillus_stearothermophilus_Bacillota

MSEGKRTVVIIGGGITGLAAAYYLQKAAKEQHLPVTCKLVEATHRLGGKVQTVVRDGFVIERGPDSFLAR

KTSAFRLVKEVGLEREIVHNATGKSYILVNGKLHPIPGGAVMGIPTRIAPFVATGLFSPAGKLRAALDFV

LPPVKTAGDMPLGRFFRRRLGDEVVDNLIEPLLSGIYAGDIDEMSLMATFPQFFQLEQKYGSLVRGAKRT

TPKEQKERKGAFQTLKTGLQSLVEEVEKRLEPGSVVKGARVERIVRVGSAYQLRLSNGETWEADSVIVAV

PHHVVPDMFADYPFFAPFRSMPLTSVATVALAFPEEAIRQDIDGTGFVVSRRSDYTLTACTWTHKKWPHT

APPGKALLRGYVGRPGDEEIVDQPDDEIVRVVLDDLSKVMKIDGRPEFAVISRWKRAMPQYTVGHRERLA

KIKQQMAAELPGVFLAGSSYEGLGLPDCIDQGEKAVGDVLDYLRQTAADERTDC

>**WP_027409017.1**_Anoxybacillus_tepidamans_Bacillota

MSDKKKTVVVIGGGITGMAAAYYLQKAIREQQLPIECKLIEATHRLGGKVQTVIRDGFVIERGPDSFLAR

KQSAFRLVREVGLQDQIVHNATGKSYILVNGKLYPIPGGSVMGIPTKIRPFVMTGLFSPIGKLRAAFDFI

LPPTKVEGDLSLGQFFRRRLGDEVVENLIEPLLSGIYAGDIDQLSLMATFPQFFHVEQKHGSLVLGTKRT

TPKQENQKHKKGIFQTLTTGLQSLVDEVEKRLEPGSVLKGIRVDKIERHHTNYRLILSSGDVWEADSIVI

ATPHQTVPGMLPDYSFFEPFKAIPSTSVATVALAFPENAIEQDIDGTGFVVSRRSDYTITACTWTHKKWP

HTAPKGKALLRCYVGRPGDEAIVEQSDDEIVNVAMDDLNKIMRINGRPEFAIVSRWKEAMPQYIVGHKER

LEAIKSKMAKQLPGVFLAGSSYEGLGLPDCIDQGEEAVKKVLGYLQVNKQTAIEVN

>**WP_023615388.**1_Mesobacillus_boroniphilus_Bacillota

MKEKKEVVIVGGGITGLAAAYYLQKHARENRLPLDVKLVEASHRVGGKMQTYVKDGFVIERGPDSFLERK

ESAGRLAREVGLGDKLVNNSTGKSYVLVKDKLHPMPGGSVMGIPTQIGPFVTTGLFSWPGKFRAAGDFFM

PPSKVKGDQSLGEFFRRRLGDEVVENLIEPLLSGIYAGDIDNMSLLSTFPQFYQVEQKYGSLILGTKKTT

PSAKMKPAETGKAKKKGMFLTVTSGLQSFVDAIESKLEPGSVIKGIRVDKVSKQANGYRMRLSSGETLDA

DSILVSAPHEAALHMFSDHEHIFDPFRNMPSTSVATVAMAFPESVIKEDIDGTGFVVSRNSDYTITACTW

THKKWPHTTPEGKVLLRLYVGRPGDEAIVELSDDEIIKIALEDLNKTMDIQAQPEFAVVSRWKEAMPQYT

VGHKERVANLKNNLADELPGVFVGGSSYEGVGLPDCIDQGEAAVAKILDYLKLN

>**WP_129726613.1**_Ectobacillus_funiculus_Bacillota

MGQKVVIIGGGIAGLTAAYYLQKHIREQQLPMETLLIEASGRLGGKIQTVRRDGFVIERGPDSFLERKTS
ASRLAKEVGLEDALVNNATGKSYVLVNDKLHGIPSGSMMGIPTQVTPFVTSGLFSPLGKLRAGFDFILPR
SKEVSDQSLGQFFRRRLGNEVVENLIEPLLSGIYAGDIDQMSLMATFPQFYQVEQKHGSIVRGMRTLAPK
TPPGAGKKKGIFLTLEAGLGSLVEAMENRLEAGTVMKSTRVEKINKTEQGYHLMLSNGKDIEAAAVIIAT
PHKMLPALFSKYREFQFFRNIPSTSVANVALAFSEDAIQKDIDGTGFVVSRNSNFTITACTWTHKKWPHT
TPKGKVLLRCYVGRPGDEEIVVRPDEDLIRTVLTDLKKTMDIDAEPEFTVVSRWQEAMPQYTVGHKERMA
KLYEFMERELPGVYLAGSSYAGAGIPDCIDQAEAVVEKVLNGIQKEFAVSV

>**WP_031768819.1**_Staphylococcus_aureus_Bacillota
MTKSVAIIGAGITGLSSAYFLKQQDPNIDVTIFEASNRPGGKIQSYRKDGYMIELGPESYLGRKTIMTEL
AKDIGLEQDIVTNTTGQSYIFAKNKLYPIPGGSIMGIPTDIKPFVTTKLISPLGKLRAGLDLIKKPIQMQ
DGDISVGAFFRARLGNEVLENLIEPLMGGIYGTDIDKLSLMSTFPNFKEKEEAFGSLIKGMKDEKNKRLK
QRQLYPGAPKGQFKQFKHGLSSFIEALEQDVKNKGVTIRYNTSVDDIITSQKQYKIVYSNQQEDVFDGVL
VTTPHQVFLNWFGQDPAFDYFKTMDSTTVATVVLAFDEKDIENTYDGTGFVIARTSDTDITACTWTSKKW
PFTTPEGKVLIRAYVGKPGDTVVDDHTDNELVSIIRRDLSQMMTFKGDPEFTIVNRLPKSMPQYHVGHIQ
QIRQIQAHIKQTYPRLRVTGASFEAVGLPDCITQGKVAAEEVIAEL

>**ARQ07576.1**_Macrococcus_canis_Bacillota
MKKCAIIGAGITGLSAAYYLGQESPEIEIDIIEASDVPGGKIKTYRKDGYTIELGPESYLGRKKIMTELA
ADIGMKDDLVVNNTGQSYIYAKNKLYPIPGGAILGIPTELRPFITTRLISPIGKVRAAMDYIIKPKPMNP
DVSVGAFFRRRLGNETLENLIEPLMSGIYGADIDKLSLKSTFPDFKKTEERHGSLIKGMRHNRKGAKSAP
KQGQFRQFKNGLQSFIERLEEVLVSRGVTIKYNHKVEAINKTSQGYEITMNGETQTYDSVIVTVPHTVFK
DWFNDGPLDYFQTMKTTSVATVVMAFDESQVNNKANGTGFVIARNSDTVITACTWTDKKWSHAAPKGKTL
LRAYVGRPGDTVVQDNDDAAIVKLARQDLDKMMEIKGEPEFSIVTKLYNSMPQYEVGHIDKIHAIQHYVA
THYPDLVITGASFEAVGLPDCIAQAQKAALKIAHR

>**WP_094905320.1**_Salinicoccus_roseus_Bacillota
MKKVAIIGAGITGLSAAHYMSKQEDVEVDLFEQSDRAGGKIRTHQQDGYTIELGPESYLARKKILTELAE
EIGLGDDLVRNQTGTSYIYVNKKLSPLPKGAVLGMPTELVPFLTTDLISARAKLRAGLDFIKKPIKVHTD

ISVGEFFRSRLGDEVLENIVEPLLSGIYSTNIDELSLLSTFPNFKDMEEEHGSLIKGVYHQKNSAPKTAP
GKKQGQFLQFRNGLQSFIDRLLEVIRENGVSVNFNHDVTAIDEEDGKYTLTVNGETRTYDNVIVTTPHFQ
YKRWFEDMPLEYFKHMKATSVATVVMAFDDHQVKNEIDGTGFVVSRKMDTSITACTWTNKKWAHAAPEGK
TLLRAYVGRPGEYIVQEKDDDEIVRLARKDLDKIMTINGEPEFTIVTKLKHSMPQYKVGHKEMIRGIHEY
MAEHYPGVVLTGASHSGVGLPDCVRQAKEAVESITE

>**WP_099693183.1**_Sporosarcina_sp_P26b_Bacillota
MSERKKVVIIGGGITGLAAAYYMQKDAREQGLPIDITMIEASTELGGKIQTVRRDGYVIERGPDSFLIRK
QSVDQLAADLGIADQLVRNATGQAYIFLHNQMHKIPAGAVMGIPTEVKPFIASGLFSLSGKLRAAGDLVL
PRSGITGDQPLGKFFRRRFGTEVVENLIEPLLSGVFAGDIDHMSLESTFPQFYEVEKKHRSLILGMKKSQ
PKQLPQKNSHSSKKVGVFHTFKYGLESLVEALEQQLTDVTIMKGTRVTKIKKHGEQAVLTLNDQQQIEAD
AVIMATSHTVASRLFEPHGLLKELATILTTSVATVALGYPKDAMKKDIDGTGFLVPRSSDHSITACTLLN
RKWPTTTPDDKVMVRAFVGRVGEEAIVDLPDAEIEKIVRKDLGDILQLEGEPEFCIITRWKDDRPQYRVG
HKEKVLRAREELHNEFPMVQLAGASYKGVGLPDCIDQGVVAKDQVIASLFN

>**WP_208143720.1**_Mammaliicoccus_fleurettii_Bacillota
MTKRIAIIGAGITGLSAAHYLKKEQPDVEIDVYEQSDRVGGKIKTYRENGYTIELGPESYLGRKRIMTEL
AEEIGIADDLVTNKTGQSYIYSHNKLYPIPGGSIMGIPTEIKPFLKTKLISPVAKLRAGMDYFLPVKPMN
TDISIGHFFRQRLGNEVLENLIEPLMAGIYGANIDKLSLKTTFPHFKEQEEEEGSLIKGMIAQKSKNNAN
KKVTVKPKGQFKQFKHGLESFMITLRNDLESKGVNIHLNSQVQNLNEENAKPILTINGVNHEYDGVIVTV
PHQHFVKWFEDDLKLDYFNYMPSTSVATVVMAFDKEQVVNDKDGTGFVIARTSDTAITACTWTNKKWPHT
TPEGKVLLRAYVGKPGSNIIHHKDEEQLKQVALNDLNKIMTINGEPEFSIVTKLPYSMPQYEVGHIDRIR
DIQQHLEKNYNNLIITGASFDAVGLPDCVQMAKDASDKMIEYLK

>**WP_127530378.1**_Paenibacillus_kobensis_Bacillota
MTTIVIIGGGVTGMSTAYYLNQYTRGAQSPVRLVVVEGSRELGGKIRTTRQGEYIMESGADSVVSRKTNM
DPLLEELSLQEELVYNATGKSYIHVDGELKLIPKDSLFGIPMTIESLVSSTLVSTEGKVEALKDFYTPNT
SFTKQDSIGRFLEAFLGKELVEKQIAPVLSGVYSGDLMELTIASTLPYLLDYKNEHGSIMKGLEANRSIL
QKGGDRKFYSFENGLSTLIDRMEERLAGEVEFRKGVRAERIDRMDDRYGVILANGETIEADHIVLGTLHT

AAQSLLGAEQLNADFAELRNSSLISVYIGFDVPDGRLPADGTGFITADSSKLVCNACTWTSRKWEHTSAS

SRLLVRLFYKSSKPAYEKLSRMTDAELLTVAVQDVKDAMGIDAAPIYYDVTKWNDTMPNYHIRHPQIVAS

LESKMEQLFPNVLLAGCSYYGVGIPDCVANGERTARRIADRITLA

>**MBT2688947.1**_Bacillus_sp_ISL_47_Bacillota

MQGERQKVVIIGGGITGLTAAYYLQKEAKEKGMLLDVKLVEASHRLGGKIQTIKRDGYVIERGPDSFLAR

KQSASRLAKEAGMEKELIRNASGKSYVLVRERLYPMPGGSIMGIPTQIAPFITTGLFSPAAKARAAADFV

LPRSNPAQDQSLGQFFRRRLGDEVVENLIEPLLSGIYAGDIDQLSLMSTFPQFYQVEQKYRSLILGMKKA

TGSQQKKAGSPKRKEGMFLTLKSGLQSLVDAIENRLEPGSVLKGYRVESIGKNGSGYTIAVNGGEELQAD

SIVMAAPHQTLHSIFSHWGLFDQFIEMPSTSVATVALAFPEEAIAKDIDGTGFVVSRNSDYTITACTWTH

KKWPHSTPKGKVLLRCYVGRAGDEAVVDLSDDQIVKIVLDDLNKTMNITMDPDFAYVTRWKDSMPQYTVG

HKERIESAKKQAKEKLPGVFLAGSSYDGLGVPDCIDQGEAAVQSVLDYLYSTKKEAVHS

>**WP_151679979.1**_Weizmannia_acidilactici_Bacillota

MKSVAIIGGGITGLTALYELQKQIKRAGAKVSLTLVEENPELGGKIRTIRHGEFTMETGADSIVARKKGV

AAFLDELGLQNRIRHNATGISYLFREDGLKPIPAETVFGIPASLDALFRSELVSTKGKLVALKDLFTKNQ

SFTKDSSAGEFLEAFFGKELVEKQIAPVLSGVYSGNLHELTLATTMPFLVEYKNQYGSIIKGLSEHKQAF

LSPERKKFVSFENGLSEIIGRLEQKSDGAEILKGIKAVALERRENGYVVALSDGRKLEADVVVLALPHAA

AQLLLRNAALDEEFNKLKTASLISVYLGFNVPDAVLPNDGTGFIVSEDSGLMCDACTWTSRKWPHTSKNG

RLLLRLFYKSTDPEWFGRLNAMDKDALIKTAMKDVEKALGVTGKPVAAEVTKWQNNMPKYDLNHRKTIEA

LTGKLAESDPNVLLAGCSYYGVGIADCIMNGKKTAEKVIRKCLLEAVN

>**WP_230575048.1**_Bacillus_rhizoplanae_Bacillota

MKKKVVIIGGGISGLTTAYYLQKEIREQGLPIDTMLIEASGRLGGKIQTLRKDGFTIERGPDSFLERKES

AARLARELNLGDELVNNATGKSFVLVNNRLHGIPSGSMMGIPTQIKPFVFSGLFSPIGKVRASFDFVIPR

SKPISDQSLGQFFRRRLGNEVVENLIEPLLSGIYAGDIDQMSLMATFPQFYQVEQKYRSISLGMRTLAPK

KPKDAKAKGIFLTLKTGLQSIVDAIEERLEDETVMRGTRVEKITKIADGYSIMLSNGKEIEADAVVMAAS

HKLLPAMFSQYKQFRSFRNIPSTSVANVALAFPKEAIQRDIEGTGFLVSRNSDFTITACTWTHKKWPHTT

PEGKVLLRCYVGRPGDEAIVEQTDEEMVRVVLEDLQKTMDITAEPEFAVVSRWKDAMPQYTVGHKERMET

LKQFMNEELPGIYLAGSSYAGAGLPDCIDQGEAAVKYVLSYLEQTNQKELMAQ

>**PEA54224.1**_Bacillus_pseudomycoides_Bacillota

MKKKVVIIGGGISGLTTAYYLQKEIREQNLPIDTMLIEASGRLGGKIQTIRKDGFTIERGPDSFLERKES

AARLARELQLGDQLVNNATGKSFVLVNNRLHSIPSGSMMGIPTQITPFVFSGLFSPIGKVRAGFDFVLPR

SKPVSDQSLGQFFRRRLGNEVVENLIEPLLSGIYAGDIDKMSLMATFPQFYQVEQKYRSISLGMRTLAPK

QPKDAKAKTKGIFLTLKTGLQSIVDGIEERLEDGTVLKGTRVEKITKIDDRYSITLSNGKEVEADAVVMA

ASHKWLPSMFSQYKQFHFFRSIPSTSVANVALAFPKEAIQRDIEGTGFVVSRNSDFTITACTWTHKKWPH

TTPEGKVLLRCYVGRPGDEAIVEQTDEDMIRVVLEDLQKTMDITAEPEFTVVSRWKEAMPQYTVGHKERM

KTLKEFMNEELPGIYLAGSSYAGAGLPDCIDQGEAAVKYVLSYLEQTNQKELMA

>**WP_025618860.1**_Salinispora_cortesiana_Actinomycetota

MTTPWRIAVVGGGIAGLAAAVQLRDHAPAGTEVTVYERSDSLGGKLHTGELAGAPVEFGAEAFLMRDSAG

GESAAVALIRRVGLADNIVHPTVGQAALLVGGELHPLPRGTLIGVPGDLAAVAAVARPTAEADVDTGQPL

LAPGVDVTVGELVRTRLGDEVVDRLVGPMLGGVYAGRADKLSLAATMPALTRTARVEHTLVGAVRAAQAA

APRSPGTPFFGTLVGGLSTLVEAAASASGATIRRNATVRALTPTGAGWHLTVGPNGAAEQVHADAVVLAV

PARPAGRLLADVAPAAAENVGDLAYASVALVTLALPATELPALSGLLVPVGEGLLMKASTFFTTKWGHLR

RPDGLTLVRASVGRYGDEAQLQRPDRDLVDTVHRELSTVLGAELPTPVATHVQRWGGSLPQYAPGHLERV

ASARATLREQRPTLALAGAGYDGVGIPVCIRSGAAAADEIITALKGSGI

>**WP_013284821.1**_Micromonospora_aurantiaca_Actinomycetota

MVRPWRVAIVGGGITGLAAAVRLRDRAPEGTEITVYEQSGRLGGKLHTGELAGGPVEFGAESFLMRDPAG

GESAAVTLARRLGLDASIVHPTVGQAALLVDGTLRQVPGGTLVGVPGDLDKVAAVAPPAADADRDAGHPL

LAEGEDTSVGELVRKRLGDAVVERLVDPMLGGVYAGRADDLSLVTTMPALARAARAEHTLVGAVRAAQAA

APRAPGAPVFGTLAGGLSTLVEAAAAASGATIRRGAAVRELRRTPDGWRLTVGPTRDAEHVEADAVVLAV

PARPAARLLAAAAPEAAGTVGALDYASVALITLALPGPALPELSGFLVPAGEGLTIKASTFFTTKWGHLR

RPDGLALVRASVGRYGDETSLQLTDDDLADTVHRELSAVLGAPLPAPVARHVQRWGGALPQYTPGHTARV

AAVRTALRAAHPTLAVAGAGYDGVGIPVCVRSGETAAEEIITALEGSAA

>**WP_010934279.1**_Corynebacterium_diphtheriae_Actinomycetota

MRIAIIGAGLAGLTAAYRLRDHDVSVFEATDRIGGKLHTVAFQSGPTDMGAEAYLAFREDTTAFFKELGL

EVVSPSGLPSLLYVNGELHPMPMNTVMGIPGSSKGLEKLVSAETAARIDAESDSMDWPQEVTLGEVLRER

FGDDLVDHVVSALQGGVYSSTSDDLSLRDTVPQLARALDRLKEEGKPVTITAAVQSIMADREKRNKARGY

KPSVFAAFPGGYAELYEALAEKSGAKIHIDAFITGIQWKDGFTLKGAEGTFDKVIVAVPAPTAAMLLKSV

APEASALLKNIKLASSAVVGMKFDSAEGLPDNSGILVATDAEVTAKAFTFSSKKWPHLGQRGGALVRASF

GRFGDDAIARADEDLLVDTALDDLQTITGFDGRAAGLSEIYVQRWFGGLPVYGPGHSDLVAAIKDALPVG

IYATGAWADGVGVPAVIDSATKVAKLLEDH

>**WP_048588526.1**_Corynebacterium_pseudotuberculosis_Actinomycetota

MRIAIIGAGLAGLTAAYEIHKLAPHADVEVFEATDRIGGKLFTVPFVSGPTDMGAEAYLAFREDTTEFFK

ELGLEESLRSPSGLPSLLYQGGSLHSMPSNTIMGIPGTSEDLGNLVSEETRKRIDSEINAESIEWPQEIS

LGALLRERLGDDVVDHVVSALQGGVYSSTSDDLSVRATVPQLARALDKLKEEGKPVTITGAVQRLLSERE

ERNKKRGYTPSVFASFAGGYAEMYEALAERSEAKIHIDAFITAVDKKPEGFVLKGAEGAFDKVLLTVPAP

TAALLLKNLVPEAAQQLKTIKLASSAVVGMKFDNSAGLPDNSGILVATNAQNVRAKAFTFSSKKWPHLGE

RGGALVRASFGRFGEDAIARADEDELVDAALDDLQTITGFDGRAAGLSEIFVQRWFGGLPVYGPGHLDTV

AAIKKALAQVEGIDATGAWADGVGVPAVIDAAKAAAKRALA

>**WP_192911964.1**_Janibacter_indicus_Actinomycetota

MPPSVVVVGGGLAGLATAHHLLRSHPDLEVTVLDGGDRPGGKVRREEIGGVRVDVGAESVVATSVAAREL

VTGLGLADRIVHPEPVPASIWSRGRRHPVPARTFMGVPGHDCDPSGLLDEGEVARAAQPTPFVVDRDDVT

VAEAVATVHGRAVVDRVVEPLLGGVYAGRVDRLSLRATMPLLWAALADGRSMTEAVDGLLPPPGTPPRPR

VMGLRGGIGGLADALADAVVAAGGRIRTGSLVRRLQRTPTGWQVIAGATTAPVVHDADHVVLATPATPTA

RLLAGHAPAASSALAGIEYASMAVMTMALPAATTPQLPGSGFLVPAVEGKAIKASTFSASKWAWVREAGG

EVVLLRASAGRAGEVASLQRDDDELVAAALAEIGEAVGSPLPTPVDHHVQRWGGGLPQYDLGHTERVAAV

RAAVADLPGIEVTGAAYDGVGIGAVLTGAAATATTILDALPPSSTPRQEHR

>**WP_159899505.1**_Ornithinibacter_aureus_Actinomycetota

MLSAALSTRPRVVVVGGGIAGLSGALAVLEALPSARVTILEGSTDLGGKLRLDAVGGHLVDVGAESMLAV

RTEATDLVARVGAEGELVTPATTSASIWSRGSLRPMPAATLMGVPTDPASALGVLTAAEVDRLRDEQPWP
GGAVESDVCVGDYVGARLGSAVVDRLVEPLLGGVYAGHASRLSLQATMPVLWERATRSESLLTPRTGATA
SDVGGANASALAQPARPPRPPFAGLRGGVGRLPGLVADELHARGALVRTGAMVRALERTASGWRLTLGSA
ADPQVIDADAVLLCVPPAPAARLLAPHAPVAARVLGDIETASSAVVTLAVERRDLAQVPGSGFLVPPIEG
RAIKASTFSFRKWAWTGEVSDVVVHLRASLGRAREEAVLHRDDADLVAVSVAEVGEALGRPLPRVVDAHV
QRWGGGLPQYAVGHVDAVASVRADVARLPGLEVAGAAYDGVGIPAVIASATRAARAALTHLSTIPSTR

>**WP_147065996.1**_Knoellia_locipacati_Actinomycetota
MTPESSTGPTVRTVVVIGGGVAGLTAARDLLVRRPGTRVVVLDASDRVGGKLRRESVGGHLVDVGAEAML
AVRHEAVDLLAELADPSDVVSPTTTSARVWSRGALHALPRTRIGVPYADSDVLGLLTDEEAARMRAESPA
PEVGSDISVGDYVASRLGDAVVDRLVEPLLGGVYAGRSRELSLRATMPAVWATATAGGSLLASPPVDPQA
GATRPPFVGLRGGVGRLPELLAADVERRGGVIESGVVARGIERTPDGWSVLTGPTTQPRLVEADAVVVAV
PPPAAARLLGPVAPGAASVVGGIEMASVAVITLAVPAAQVASWPGSGFLVPPVDGRGIKASTFSSAKWGW
LAAEGADTAYVRASVGRAGENATLQRDDTDLVALAALEIAEAVGAPPLTVVDSHVQRWGGGLPQYTVGHV
DRVARVRAALAEVPGIEVAGAAYDGVGIPAVIASARLAADATATHLERPAGRAGE

>**CNE49003.1**_Mycobacterium_tuberculosis_Actinomycetota
MTTSHAVVVGGGIAGLAAARVLARDGVRVTVLEGSPRIGGKLRVGDIAGIPVDEGAESMLARRPEGMDLV
RDLGRSGELVNPGTTSSAILSRGVLRTMPAGQVMGVPSDLRALAASHVLSPAGLARVPLDLVLPETPRGG
DVSVADFVGARVGREVVDRLVEPLLGGVYAGRVERLSFEATLPQVAAAARGHRSLIGAVQGLRAAAPKDP
GPVFATLPDGLGTLPGLVAADITAAGGTVRTGATVRELRRTPDGWRLTIGSTRDPEYLDADAVVIAVPAA
PASRLLEEEVPAAARELAGIEYASMAIITLAYRATAFPRLPKGSGYLVPGVESDTERGGRGVKAVTFSSV
KWPHLRRRDRGVIAVRCSIGRFGAEHTLQRPDEELAATAMAELAATCGVTELPAESRVTRWGGGLPQYNV
GHADRVAKVRSAVAAAPGLAVAGAAYDGLGVPACIASARAAATRVLDHLRSREGAHHDRAGGQAEGTRTQ

>**WP_185025794.1**_Actinomadura_coerulea_Actinomycetota
MTTSHAVVVGGGIAGLAAARMLAREGVRVTVLEGSPRIGGKLRVSEIAGIPVDEGAESMLARRPEGLDLV
RDLGRSGDLVNPGTTSSAILSRGELRPIPSGQVMGVPSDLKALAAAHVLSPAGLARVPLDLVLPETPRGG
DVSVADYVGARLGGEVVDRLVEPLLGGVYAGRAERLSFESTLPAVAAAARSHRSLIQAVQGIRDAAPKNP

GPVFATLPDGLGTLPHLVAADIAEAGGTVRTGATVRELRRRENGWRLTVGSARDPERLDADAVVVAVPAA
PASRLLEPDVPAAARELAGIEYASMAIITLAYRATAFPGLPEGSGYLAPNVESDTARGGRGVKAVTFSSL
KWPHLRDRDPGVIAVRCSIGRYGEEHLLQRTDEELTATAMAELAATCGVTELPAESRVTRWGGGLPQYNV
GHADRVAKVRAAVAGTPGLAVAGAAYDGLGIPACIASARAAALRVLDHLRSREGVGP

>**WP_189181473.1**_Microbispora_rosea_Actinomycetota
MDGRAGRHVVVVGGGIAGLAAALHLNRADAGLRITVLEAAPRVGGKLYASQVAGVEVDAGAESMLARRPE
GKELARLAGLGDDLVSPGTTRAAVLSRGSLRPLPRQQVMGVPSDVVALARSGILSPGGLARVPLDQFLPP
TLVTGDVSVAAYIRARMGSEVVDRLVEPLLGGVYAGRADMLSLDATMPRIAAIARTERSLLQAVRGIASS
APADAGPVFASLRYGMGSLPAAVAKASGALIRTGVTVRDLLRTPDGWRLVTGSTRDEETIDADAVVLAVP
ARPASRLLRREVPKAASELAAIEYASMAIITLAYPRTAFPEPPEGSGYLVPPVEGRPVKAATFSSVKWPH
LAEAGRDVVIVRCSIGRLGEELVLQRDDSELTALAMNEMAEILGVRGLPLDTRVTRWGGSLPQYDVGHLD
RIARVRAALAGQPGLAVCGAAYEGVGIPACIATARTAAKLVIEHLRDKRAAAASGA

>**WP_109279126.1**_Streptomyces_orinoci_Actinomycetota
MNAAHPHRPRVAVIGGGISGLAAAYRLVTGGAEVTVLESETRLGGKLLAGEIAGVPVDLGAEAMLARRPE
AVELARAVGLADSLQPPAAAGARVWTRGGLRPLPQGHLMGVPGDLAPLAASGVISEAGLARMARETELPG
TELGEDVAIGALVAERFGREVVDRLVEPLLGGVYAGDAYRISMRAAVPALFAAARRHPSLTEAVRAVQAA
APARGGPVFMGIDGGVGRLPLAVAEAVRRAGGEIRTGAPVRALTRLPGGWRLDLGDAYLTADAVVLATPA
GAAARLLDTTAPAAAGELAAVEYASMALVTLAFRRSEVAHLLDGSGFLVPPVDGRKIKASTFAANKWGWI
ASADPDVFVLRTSLGRYGDEADLEREDAELVDLSLKDLGEAVGLAARPLRSRVTRWYGGLPQYPVGHLRR
VARIRDAVAALPGLRVCGAHYDGVGIPACIAAAHRAADELLATLAEGTAGEGGE

>**WP_119731172.1**_Thermomonospora_amylolytica_Actinomycetota
MSERRSPRPHVAVVGAGIAGLTAAWRLVRGGARVTVLEGSPRIGGKLQVSEVAGIPVDEGAESLLTRRPE
GVELLRDLGLTGERVHPGGVASAIYSRGGLRSLPAGQMMGVPGDLRALAASQVLSPAGTARAALDLVLPR
TPLGADVSVAAYIGARFGGEVVDRLVEPLLGGVYAGRADLLSFEATLAQVAAAARTHRSLVTGVRALRGA
GPRDAGPVFTTLPDGLGALPGLLAGALGEDCEIRTGAMVRELRRLGDGGWRLTFGPARAPQTLDADAVVV
AVPAQPAARLLAAEVPDAARELEAIEYASMAIVTLAYPATAFPRRPGVSGYLVPAVEERQVKAVTFSSVK

WPHLTERAPDLVIVRCSIGRYGEEHTLQRGDEELRAAAIAELAAVCGARELPVDSRVTRWGGGLPQYTVG
HVDRVARIRAAVAAVPRLAVAGAAYDGLGVPACIATARAAADRVLEQCESRGGTDHDNGQRGQADAGRQG
ARP

>**WP_141788351.1**_Oryzihumus_leptocrescens_Actinomycetota
MASTRVAVVGGGISGLAAAWQLVHDLPGAEVVVLDASDRPGGKLRSAVVGGVRIDVGAESMLARRPEGLD
LATEIGLAGALAHPATTRASVWSRGALHPMPGGTLMGVPANPAAALGLLDAEEIARDEQERDLPVTPLTQ
DVSVGDYVAERVGPAVVDRLVEPLLGGVYAGHARLLSLQATVPALWQAAVRGEPLTVAAERAASAAAANG
TPVFAGLRGGMATLAESLHAALVARGVRVVSDTIVRSLERTPGGWRLVTGPVPAPVALDVDAVVLSVPAA
PAARLLAPHSAPASTELAGVDYASMAIVSLVLPRAGMPPMPGSGFLVPPVEGRTIKASTFSSAKWGWVAE
AAGDLVVLRASIGRHGEEADLQRADQDLVAVARKEVGEALGGRLPAPVDAHVQRWGGALPQYAVGHVERV
ARIGEAVAGLPGLEVAGAAYQGVGIPACIASGRGAALAVATHLQDRVTGGGE

>**WP_189646136.1**_Nonomuraea_spiralis_Actinomycetota
MEGNRAHVVVIGGGISGLAAAWFLRQGTSERVKVTVLEGSPRLGGKLHASEVAGVSVDAGAEAMLARRPE
GVELAKAVGLGDELVFPGTVRSSVYSRGALRQMPKGHVMGVPADLAELARSGIVSPGGLLRVPLDQVLPP
TLVRTDVSVASYIRARMGGEIVERLVEPLLGGVYAGRSDMLSLEATMPQVAAVARAERSLLSAARHIVEG
TPKNAGPVFTTLRQGLGGLPEGVAKASGADIRTGVTVRELHRTEHGWRLVAGPVPQPEVIEADAVILATP
GPAAARLLKAEVPKAAAELARIEYASMAVVTLAYPLDAFPQPPTGSGYLVPPVEGRPVKAVTFSSVKWPH
LGQDLLIVRCSVGRIGEEHLLQREDAELVSLAMAELDEVVGVRGLPVDSRVTRWGGALPQYNVGHLDRVA
RVRAAVSGVTGLAVCGAAYDGIGVPACVGTARTAAARILDHLSQRGE

>**WP_063813067.1**_Herbidospora_daliensis_Actinomycetota
MVMERFHVVVVGGGIAGLAAAWHLRNDRIRVTVLDGAARIGGKLVATDVAGVPVDAGAEALLARRPEGVE
LARLAGLGGDLVDPGTTSASILSRGELRPMPKGQVMGVPSDLVALARSGVLSPAGLARVPLDQVLPATLV
TTDVSVGSYIRARMGDEVVDRLVEPLLGGVYAGFADRLSLEATMPAVAAAARSEASLLRAVREITGAAPQ
NPGHVFTSLKNGMGSLPPALAAASGAQVRTGVMVRELSRTPSGWRLTTGPTRDEHTLDADAVVVAVPATP
AARLLDREVPKAAAELARIEYASMAIVTLAYPIEAFPQVPSGSGYLTPPVEGRAVKAVTFSSVKWPHLGG
HGYVFVRCSLGRLGEERLLQRDDAELVALAMNEMTEIMGVRGLPADTRVTRWGGALPQYDVGHNDRVARV

RAAVAAVPGLAVCGAAYDGVGIPATAATARMAAARILDHLDPRAEWRHDGRHPAAQGA

>**WP_051712293.1**_Spirillospora_albida_Actinomycetota

MTTSHAVVVGGGIAGLAAARVLAAAGARVTLLEGSPQVGGKLRVGDIAGLPVDEGAESMLARRPEGIGLV

AALGRAETLVHPGTTASAILSRGELRRIPSGQVMGVPSDLRALAGSQILSPAGLARVPLDRILPETRRGD

DVSVADYIGARMGREVVDRLVEPLLGGVYAGRAEELSFDATLPAVAAAARTHRSLLAAVRGVRDAAPHGA

GPVFATLPDGLGTLPHLVADAITAAGGDVRTGATVRELRRTEHGWRLTVGSARDPEHLDADAVILAVPAP

PAARLLEHDVPAAARELARIEYASMAIVTLAYPVSAFPRLPKASGYLVPSVEVGPGGVKAVTFGSVKWPH

LRRRDNGLIIVRCSIGRFRDEAVLQRPDAELAAGAIAELAATCGLTELPAETRVTRWGGGLPQYNVGHAD

RVARVRAAVAGEPGLAVAGAAYDGLGIPACIASATAAADRIIDHLRSRGGFDHGDGAGQAEGTRAQ

>**WP_189162124.1**_Sphaerisporangium_melleum_Actinomycetota

MEATRRHVVVVGAGIAGLAAAWHLRHEAPGLRVTVLEGAPRIGGKLRVSQVAGVPVDEGAEAMLARRPEG

KELARAAGAADELVHPAPVGASVYSRGALRPMPKGQVMGVPTDLTELVRSGVLSPAGLARVPFDQILPAT

LITSDVSVAAYIRARMGGEVLDRLVEPLLGGVYAGRAEMLSLDATMPAVATAARTERSLLQAARAIAEKA

PKDAGPVFTTLKGGMGSLPERVAAASGAEVRTGVMVRELSRTPGGWRLVTGPTRDPETIEADAVILAVPA

PAAARLLSGEVPKAAAELSRVEYASMAIITLAYPRTAFPEQPSGSGYLVPPVEGRAVKAVTYSGVKWPHL

AEADPGVLVLRCSIGRLGEEAVLQRGDDELVALAMAEMAEVAGARGLPLDSRVTRWGGALPQYEVGHLDR

VARVRAAVAVQPGLAVCGAAYDGVGIPACVASARSAATAVLSHLRDEGGGEWREDRRAAGAR

>**WP_214107560.1**_Acrocarpospora_catenulata_Actinomycetota

MEKDRHVVVVGAGVAGLAAAWFLRQAGGDRLRITVLDGASRIGGKLHASEVAGVRIDAGAESMLARRPEG

LELVRGLGLDEELADPATTRAAVLSRGALRPMPGGQVLGIPGDLTALAKSGVLSPGGLARVPMDQVLPPT

LVHEDVSVAAYVRARMGEEVVDRLVEPLLGGVYAGRADRLSLTATMPAVAAAARTEGSLLKVARDLVAAT

PKNAGPVFTTLRDGMGSLPAAIAEASGARVRTGVMVRELSRTATGWRLVTGPTRDAAVVQADAVVLAVPG

PAAAKLLGKEVPAAARELAAVEYASMAIVTLAYPREAVPGLPEGSGYLVPPVEGRPVKAVTFSSSKWPHL

AEHGLLLARCSLGRIGEEYVLQREDADLVELGVAELAEVAGVRGRPIDTRVTRWGGGLPQYNVGHTERVA

RIRAAVAGQPGLAVCGAAYDGLGIPACVGSAKSAASRILDHLDPERKWSHDDQPATQGA

>**WP_203944764.1**_Planotetraspora_thailandica_Actinomycetota

MDGTSGRHVVVVGAGIAGLAAAWHLSRADPGVEITVLDGASRIGGKLYASEVGGVQVDAGAEAMLARRPE

GKELARQAGLGEALVHPGTTQAGILSRGSVRPLPKGHVMGVPSDVMALARTGILSPGGLARVSLDEVLPP

TTVPTDVSVGAYVGARVGGEVVDRLIEPLLGGVYAGRADMLSLDATMPAVAAAARTERSLLRAARDLAAK

TPKDAGPVFTTLRQGLGGLPEAVARASGARIRTGVMVRGLTRTPDGWRLVTGPTRDEEVVDADAVIVAVP

ATPAARLLEREVPKAAAELARIEYASMAIVTLAYPRSAFPEPPAGSGYLVPSVEGRPVKAVTFATTKWPH

LAEADPNLVIVRCSIGRLGEEAVLQRDDAELVAFAMNEMTEIMGVRGLPIDSRVTRWGGGLPQYDVGHLD

RIARVRAAVAAQPGLAVCGAAYDGVGIPACAASGRTAATRVLEYLNARAAAGARQEPGD

>**WP_012893159.1**_Streptosporangium_roseum_Actinomycetota

MEGNRCHVVVVGGGIAGLAAAWYLRQGGEGVRVTVLDGARRVGGKLLATEVAGVSADAGAEAMLARRPEG

KELARMVGLGEELRHPGTTQAAILSRGALRPMPKGHVMGVPSDLASLARSGILSPGGLARVPLDQILPAT

LVGTDVSVAAYIRARMGGEVVERLIEPLLGGVYAGKADRLSLDATMPRIAIAARSERSLLAATREIAAEA

PKDAGPVFTTLRRGMGSLPEAVAAASGAEIRTGVMVREMRRTESGWQLVAGPVPEPEVIEADAVIVATPG

PSASRLLAGEAPLAAAELARIDYASMAIVTLAYPREAFPRPPDGSGYLVPPVDGRPIKAATFSSVKWPHL

AEADPNLILLRCSIGRLGEEAVLQRDDAELVALAMAEMVEVMGVRGLPRDSRVTRWGGSLPQYDVGHLDR

VARIRAAVAAQPGLALCGAAYDGVGIPACVSTARTAAARILDHLDPTGEWQKRADSLTS

>**WP_194698458.1**_Nocardioides_agariphilus_Actinomycetota

MSALTRRTVVVGGGLAGLAAARELAATGRDVVVLEGSPRIGGKLLTGEVAGIAVDVGAESMLARRPEGIE

LARGLGLEVVHPATTSSSVWTRGELRPLPRSVMGVPGDLAQVADSGVLSDTGLDRLRAEQPGPAVDDADD

VSVGELVASRLGDEVVDRLVEPLLGGVYAGHARNLSARATVPQLVAWSGGSLVAAAASALAASDGSAVFA

GIPGGLGRVPGTLASGLDVRLDAPVRALHRAPDGFLLVVGPTTAPEEVRADEVVLATPAAPAARLLRDLL

PHAAAELAGVEYASMAVVSLAFRAVDLPELSGSGFLVPPVDGRTIKASTFSFNKWDWVRAAGGEVLVLRT

SVGRHREESVLQVPDDELVSASLRDLGEAVGLGATPIDTHVQRWGGGLPQYAVGHLDRVARIRSEVGRIP

GLAVCGAAYDGVGIPAVIASAHRAVVALTRAQ

>**WP_033269126.1**_Streptomyces_lydicus_Actinomycetota

MSAAHPGPGPAPTGHVVVIGAGISGLAAAHRLLDGGARVTVLEAADRPGGKLRSGEIAGVPVDLGAESML

ARRPEAVELARAVGLGDRLQPPTTATASLWTRAALRPMPKGHVMGVPGDLAPLAAAGVLSPAGLARIEED
ARLPRTEVGEDVAVGEYVARRLGREVVDRLVEPLLGGVYAGDAYKISLRAAVPQLFEAARTHRSLLDGVR
SIQARAATAAPLTGAAATGPVFMGIDGGIGTLPGAVADAVRAKGGEIRTGTPATGLRRTPDGWHLDVDGG
RLTADAVVLAVPAPAAARLLADVSAPAAGELGTVEYASMALITMAFRRADLDRQLTGSGFLVPPVDGRTI
KASTFSANKWGWLGDADPDLFVLRTSVGRHGEEQDLARDDSELVALSLADLREAVGLTAAPVASRVTRWD
GGLPQYPVGHLERVARIQDAVAGLPGLRVCGALYEGVGIPACIASARRAADDILGTLRPATVPGE

>**ANW21801.1**_Streptomyces_clavuligerus_Actinomycetota
MVVVGGGISGLAAAHRLVTAGVRVTLVEASERVGGKLHTGEIAGVPVDLGAESMLARRPEGIDLARAAGL
GDRLRPPATATASIWTRGALRPMPQGHVMGVPATPDSLAGLLSPEGVERIGRERDLPPAAVGDDVAVGTY
VAERLGREVVDRLVEPLLGGVYAGDAYRISLAAAVPQLFAAARGHGHLLDAVDAVRRETAARQADGPVFL
GIDGGVGTLPLAVAAAVRAHGGTLLTSTRALGLARVPGGWRVRTEHGVLEADGVVLAAPAWTTAELLAAE
SPVASAEIGRIEYASMALVTLAFRRSDTTGLPEGSGFLVPPVDGRTIKASTFSSRKWSWVDKGAPDLVLL
RASIGRYGEEEQLERDDADLVRAALTDLGAATGLAARPLATEVTRWIGGLPQYPVGHRARVARVRAEVAK
LPGLTVCGAAYDGVGIPACVADGRRAAEEILATPTLARGTDHGAGQ

>**CBK40973.1**_Nitrospira_defluvii_Nitrospirae
MARTPRSVVIVGGGISGLSTAFALQEQAAAAGMALTCTILDAAPVWGGKILTHRVGQLVMEAGPDSFLSQ
KPWGMELCRRLGIADQLINTNPVEKKASVLRGGQLHELPEGLVTFTPTQLGPFFRSGLLSWVDLARMGCD
VLIPPRRSTDDESLASFFRRRFGRHACERVMEPLMAGIYAGDAEQMSLRATFPRFYELEQAHGSVIRGMM
AARRARAQKVSGGGPRHTMFVTLKNGLADLVAGLTAQIQQAGGVLKAGVQAEALRVRSHQAGRWMYDVMC
TDGTAISAEALVLATPAYVSAELVRPLTPMAAGLMDMIPYASTATISLIYPAEAVGNRLQGFGFVVPRSE
GRDLIAATWTSLKWPHRAPPEDVSVRCYLGGVGREVILQRDDEALVRCVREELASIVGLQATPHYVEVNR
WNRAMPQYTLGHLDRLVQLDAALSRFGGLAVTGAGYRGVGLPDCIRDGADTAAKILHYLHTAPM

>**WP_168060950.1**_Cand_Manganitrophus_noduliformans_Nitrospirota
MGTEKKKVVIIGGGITGLSTAYFLQEKMKENGLAIDCTLIESDSRFGGKVVTERVDGFVIEGGPDSFITQ
KPWALDLCKRLGLTDRLIQTNPVEKSIYILSKGQLCSIPEGFNLMVPGRVMPFLFSPLVSLSGKARMGLD
LLIPRKETATDESIASFVRRRLGQAAVEQFAEPILAGIYAGDAEKLSMMATFPQFAQMEREHGSLVWGMW

MRRWDAAKKPPRKSEWSLFVSLRDGLAGLIEAIRSRLDQVTLLSGRKVIGVRPMEGRFEVSLDGERLLAD
AVVMTTKTHTAADWIEGWDAPLAKRLRENEYVSTATVSLGFRKADVPHPLNGFGFVIPRREKRKIMAATW
TSTKFPGRAPEGHVLIRSFLGGAHQEEVVGLDDASLVSIVREELRSILKIKAEPVVARHFCWIKANPQYH
VGHLDWVEGVEKEAAKHRGLYLIGAAYRGVGLPDCIHQGMETAEKIVRSVSSIK

>**QPJ64655.1**_Cand_Nitrohelix_vancouverensis_Nitrospinae
MKKIIIIGGGIAGLAAAFRIQEEIDNGADVQCQVLEASEHFGGKIHTERFDGFIVERGPDSFISQKPWAI
QLCKRLGLAHRLMGTNPEQPKTYVYTGQRLVTMPDGLSLMIPTKFLPFALSPLFSWSGKIRMGMDLIIPG
KKNDEDESLASFIRRRLGEEALRKMAEPMLAGIYASDPETMSIKSTFPMFVQAEKKYRSLILGALARKRD
MLMKKPATPAPKGPAPFSLFMTLQSGLGEMVDAVLEKSKDIQFRPNTRIDSITRNGEGWKVNVEGGEALE
ADIVIVSCPASITAKLIEPTAPKASELLKTIKYVSTATVSIAFKKEGFEHPLNGFGFVIPRNEGRRILAC
TWTSSKFPKRTPDDHVMLRVFVGGAQREDMAEQEESAIVTMVREELKFMMGIEQEPVFCKVYHNRKSNVQ
YQVGHGELIASIEKEMEAHPGLYLAGSAYHGIGIPDCVLDGTKAAEKALGVAPVPAP

>**WP_008476560.1**_Nitrolancea_hollandica_Thermomicrobiota
MAHAVIAGGGISGLTAAYRLRRLARESGLDLRITLVEADTRLGGKILTERVDDLIIEAGPDSILSQKLPA
IHLCQELGIADRLVGTKEGGGGTYILREGRLEPLPEGITMLVPTKLRPLIGSRLLSTRAKLRMGLDVLIP
PLQSGDDESVAAFVTRRLGREAFERMAQPLLSGIYAGDAGKLSLAATFPRLRQIEREHGSLVRGMLAQRR
QRRTASGSAQRYTPFVSLRGGIGELVDVLTAQLEDVDIRLGTAVNGIEQRTSGGYRVRLSDGETLETDVL
LLATPADVSGNLLAPVRPDLAALLQQIPYVSSATITLAYRQSEVGKLGAGRGFVIPRVENRELTAVTWAS
SKFPYRAPEGLMLLRTFVGRAGRESAVDLPDDLILRLVREELREILGLTAQPVLSRIYRWHQALPQYVLG
HLDRLAEIDRELADLPGLFLLGAAYRGVGIPDCIQSGNQAAERALALLKAQSSETSHTA

>**WP_273030691.1**_Sphaerobacter_thermophilus_Thermomicrobiota
MAAGRVAIIGGGIAGLAAAYRLRQADSGLALTLVEADTRLGGKIRTERMEGLVIEAGPDSFLASKPAAAA
LCEVLGLGDRIVSTTRESGGTYILHRGRLEPLPEGITMLVPTKVRPLLRSRLLSTRGKLRLATEYFRRPR
LDDADESVASFVRRRFGDEVFDHMAQPLLSGIYAGDAEQLSLLATFPRLRETELRYGSLIRGMLAQRRAA
PPPPPGGGQPRGAFLSLRDGLGELIDALVASLDGIDWRVGVAAVAVEPVNGGWIVHLADGSAVAADAVLL
ATPAWATADLVAGIDEELAELLRGIPYVSTATISLAYRREDVGQVGVGRGFVIPRIEGRELTAVTWASSK

FPHRAPEDLVLLRAFVGSAGREEAVDLPDDQLLDLVRRELREILGMDATPVLTYIHRWYRALPQYVLGHL
ERVEAIDRRLEQHPGLLLLGAAYRGVGIPDCIVSGERAAARALAYLAGAAEPSRQPQERAG

>**WP_015897757.1**_Acidobacterium_capsulatum_Acitobacteria
MKHTVILGGGITGLTAAYILRQRAGGAMRVTLLESGQRLGGKIATAEENGFLMEGGPDSFLARKRVTMEI
CRELGLEEQLMPTAPGERTTYVWSGGKLHPLPMGVTPMLRSKLISWPGKLRMGAEALIPPRSVEEDESLA
SFVGRRFGREALDKLAAPLMAGIYSADATRLSMQSTFAMLPAMEKKHGSVLRGWLRSKQTHKNSGAGAGT
MFLTLRGGMRQLVEALTAELPREDVRMDCPALAVLPKDGKYEVVLRGGDSLLADDVILATPAAVSARLLD
LLDPQLAARLRMVRYVSTATVSLGFRESDLAAMPKGFGFVVPRQEGRKITACTWSSMKFAGRAPEGHLLL
RVFLGGAGAEHVAEQDGAALVEAARQELRRTMGIAAEPVTARVYRWEKGTPQYEVGHAERVAEIEALAHG
HRGLFLAGAAYHGAGVPDCMQNALDIAGRLAAQYGMSAALAWNAVREQGTVTERK

>**WP_158787781.1**_Granulicella_sp_L46_Acidobacteriota
MPKTIVIGGGISGLAAAYALQKSEADYLLIEASDHLGGKIVTYAGEGFLIEGGPDSFLTQKRAALDLCRE
LGLGDQLIGSNHTATPSTYVLSKGKLHPMPEGMMLMAPTMILPILRSELISWPGKLRMGLEIFIGRNTTV
ADESLGSFVRRRMGSELLAKIAGPLMAGIHAGDPEALSLRSTFPMFTDMEKTHRSLVLGMMKRKKAQAAQ
LTTGPRPSMFTTLSGGLQQLPNAIAARLNPQQVKLNTRVQSVAAAGGQYRISLADGTSLIADNVVFTTPA
YVTAEILQQLTPALAEKLRRIRYVSTSTVSLAFRRSEITCDLNGFGFIVPAAEGRKINACSWSSTKFSHR
APDDFVLMRVFIGGAFAEDLAEQDEATLIDIARNELRDIMGITATPVLARAYRWTKSNPQYNVGHGALIK
EIDQLVSAHPGLYLAGAAYRGSGIPDCIQSGVDTAAKIVPRQTSSNPAISHAAPAFVAT

>**WP_012120536.1**_Roseiflexus_castenholzii_Chloroflexota
MTAMHSTSAATLFPGGQPHIVVVGGGISGMSAAYELGRATRDGAPPVMVTLIEREARLGGKVVTERNGPF
VIEGGPDSFMAQKPWAAELAREIGLGDELMVASPMRRTTWVLIRGRPQPLPEGMLLIVPTRIAPFAFSPL
ISPLGKLRMALDLFVPARRDDGDETLADFIRRRLGNEALDRLAEPILSGIHSAECERQSILATFPRFREL
EKRHGSLIRGMLAARRTASPSSAHQSPFMTLRGGMGTLVERLEQRLTARILTNRRVMALTCDTTAARPYR
LWLDDGATLDADAVILATPSYAAADLVGASFPALADALRAIRYVSTATVSLVYRRSEVGTPLDGYGLVIP
RSEQTWINACTLSSVKFRHRAPDEYLLLRCFVGGSRRPELLARDDDDLVRMAQSDLRAVLGITAVPLLTR
VYRWHNGNPQYDVGHLERIAALEALCPAGLLLAGAAYRGVGVPDCIKQGREAARRALDVVATARYPVMEK

>**WP_140861053.1**_Myxococcus_xanthus_Proteo_delta

MHHMPRTNGMNVAVVGGGISGLAVAHHLRSRGMDAVLLESSARLGGAVGTHALAGYLVEQGPNSFLDREP

ATRALAAALNLEGRIRAADAAAKRRYVYTRGRLRSVPASPPAFLASDILPLSARLRVAGELFSRRAPEGM

DESLAAFGRRHLGHRATQVLLDAVQTGIYAGDVEQLSVAATFPMLVKMEREHRSLILGAIRAQKAQRQAA

LPAGATPKLSGALSTFDGGLQVLIDALAASLGDTAHVGARVEGLAREDGGWRLIIEEHGRRAELSVAQVV

LAAPAHAAAKLLRPLDDALAALVAGIAYAPIAVVHLGFDAGTLPAPDGFGFLVPAVEQRRMLGAIHASTT

FPFRAEGGRVLYSCMVGGARQPGLVEQDEDALAALAREELKALAGVTARPSFTRVFRWPLGIPQYNLGHL

ERVAAIDASLQRLPGLHLIGNAYKGVGLNDCIRNAAQLADALVAGNTSHTP

>**WP_224242107.1**_Cystobacter_gracilis_Proteo_delta

MTVVAIVGGGITGLALAYRLRSRGKDVVLLEGETRLGGNIQTRRRDGLLTEAGPNSFLDKEPATRELAAA

VGVEDRIRAADPAAKARYLYTRGRLRAVPASPPAFLKSDILPLGARLRVVAELFTGRVSGEVDESLGAFG

RRHLGTTATATLLDAVQTGIYAGDMEALSVGATFPQLTKLEREHRSLILGAIRTQGAQRKALPAGGPAKL

RGALSTFDGGLQTLVDGLATMLGPAVHTGAKVEGLQPGHGGWRVSVREHGRQAELMASRVVLATPAYVTA

GLLRPLDEPLSALVEGIAYAPIAVVHLAYAPGSTPAPDGFGFLVPGLEKRRLLGAIHASTVFPFRAEGGH

VLYTCMVGGARRPDLVKLDEEALVTLAREELKELAGVTASPVFAEAIRWPRGIPQYNVGHLERVASIDAA

LARLPGLHLAGNAYKGVGINDCIRNAFALGDMLA

>**WP_203407528.1**_Archangium_violaceum_Proteo_delta

MSVVIIGGGISGLALAHGLRARGTEVTLLEAGPKLGGNIQTLSRDGFIIEAGPNSFVDREPTLRQLAASL

GIEGRIRTADPAAKRRYIYSRGKLREVPSSPPALLKSDVVPLGAKLRMMGELFTRRAPDGDESLADFGRR

HVGRTATAVLVDAMQTGIYAGDVEALSVGAVFPKVVELEKQHRSLVLGMVRTQKAARKALPPGEGAPKPS

GAVSSFDGGLGVLVDALARSLGTAARTGVRVEGLRREGNGWRLAVEEHGQRAELTADRVVLAVPAYVAAK

LLRPLDETLAARVEGIAYAPVAVVHLGFAPGSLPPPDGFGFLVPTLERRRVLGVIHASSTFPWRAEGGRI

LLTCMVGGAKRPDLVELDDAALVTLAREELRELAGVTAEPSFTEVFHWKRGIPQYNVGHLERVAAIDEGV

ARLPGLYLTGNAYKGVGLTDCIRNANALVDTLAR

>**WP_044193071.1**_Hyalangium_minutum_Proteo_delta

MTVIAVIGGGISGLTLTHCLRSRGKDALLLEASSRLGGNIETRQRDGFLIETGPNSFLDREPATRELAAG

VGVEDRIRSADPAAKARYLYTRGRLRPVPSSPPAFLKSDILPLGARLRVMAELFTGRAPEGVDESLAAFG

RRHLGPAATAVLLDAVQTGIYAGNMETLSVDATFPQLTKLEREHRSLILGAIRSQKAQRKALPAGAAGSP

EKLRGTLCTFDGGLQTLVDGLARELGPAAHTNAKVEGLQPSHGGWRVSVRENGGQAELLASQVVLATPAF

VAAGLMRPLDEPLAALVEGIAYAPIAVVHLGFAPGSTPAPDGFGFLVPGLEKRRLLGAIHASTVFPFRTE

GGRVLYTCMVGGARQPDLVKLDEEALVALAREELKELAGVTASPSFTEVIRWTRGIPQYNVGHLERVAAI

DAALKRWPGLHLTGNAYKGVGINDCIRNAFALGDALAA

>**WP_093525860.1**_Stigmatella_erecta_Proteo_delta

MAVIAVVGGGISGLALAHRLRSRGKDAVVLEAAPRLGGVIQTRHREGFSTEAGPNSFLDREPATRELAAS

LGVEERIRMADPAAKARYVYTRGALWPVPTSPPAFLKSELLPLGARLRVLAELFTGRGPADRDESLGDFG

RRHVGTQATSVLIDAMQTGTYAGDLEALSAEAAFPVLKQLEREHRSLILGQVRTQRAKRQAAPEGPKLKG

AMCTFEGGLGTLVDALAQALGPAARTGAAVEGLTRTAEGWRVAVRERGARAELEASQVVLTAPAPVSAEL

LQPLDAPLAALVKGIVYAPIAVVHLGFAPGRTPPPDGFGFLVPAQEHRRLLGAIHASTVFPFRAEGGRVL

YTCMVGGARRPDLVGLDEEALAQVAQEELRALAGVTASPDFTEVIRWPRGIPQYTVGHLERMAAIDTALA

RLPGLHLAGNAYKGVGLNDCIRNAAALGEALASR

>**WP_169825158.1**_Corallococcus_exiguus_Proteo_delta

MTVAVIGGGITGLALAYQLRARGTAAVVLESTSRVGGNVQTHAREGYVLEAGPNSFLDREPTTRELADVL

GVSSRIRSADAAAKNRYIYTRGALRALPTSPPAFLKSDILPLASRLRVVGELFSGRNPTGADESLARLGR

RHLGREATAVLLDAMQTGTYAGDPEQLSAEATFPQLVKFEREHRSLILGAIRAQRAARQAKGAGAVETGP

GLTGQMSTFDGGLGVLVDALAKALGDSVRTDAKVEGLERAADGWKVRYQERGQPAELSASHVVLAVPAHV

AASLVRPLDAELAEKADAIPYAPIAVVHLGFAPGTVPKPDGFGFLVPAVEGTAMLGTIHVSTTFPFRVEG

GRVLLTCLMGGARRPEVVSRDEDALVALAREELKTMAGLTATPELTEVFRWPRGIPQYTVGHLERLAAME

ERLKRWPGLHLTGNAYRGVGVNDCLKEAARLADALGGLAAGATRSA

>**WP_095979663.1**_Melittangium_boletus_Proteo_delta

MRVVVVGGGISGLVLAQGLHARGTDVTLLEAAARPGGNIQTHRRDGFITEAGPNSFLDREPTLRALAARL

GIEERIRTADPAAKRRYLYTGGRLRALPQSPPALLRSDVLPWSAKLRMLGEPLTPPAPGGDESLADFGRR

HVGRAATRVLVDAMQTGIYAGDMESLSVAACFPQVAALEKRHRSLLVGMVRARKEKRAAPPPPEGTPSTT
GAVASFDGGLGVLVDALAQALGPVLHTGARVVGLRREGPGWRVTVEEAGRREEREADRVVLAVPAHTAAE
LVRPLDAPLADRLAGIVYAPMAVVHLGFAPGTVPPPDGFGFLVPAAERRRVLGVIHVSSTFPWRTEGGRI
LYTCLIGGATRPDLVELDEAALVTLAKEELRLMAGVTAEPVFTEAVRWKRGIPQYNRGHLERLAAIDAAL
AHLPGLSLTGNAYRGVGLTDCVRDATARVDALTR

>**WP_217081799.1**_Citreicoccus_inhibens_Proteo_delta
MTVAVVGGGISGLVVAQQLRTDGKAVVLLEADSRLGGNVHTRARDGFLLEAGPNSFLDREVVTRRLTTAL
GITGRIRAADVGAKNRYVYTRGQLRAVPGSPPAFLKSDVLPWSAKLRVMAELFTPSRPNAEDESLAQFGR
RHLGRTATRVLLDAVQTGIFAGDVETLSVEAAFPALVKLERAHRSLILGAIRSRGAKQKALPSGASEPLL
SGALSTFDGGLQVLIDALSTALGDVARTNAQVLALVPMPNGWKLIVREKGETSELLASQVVLAVPAHVAA
ELLDPIDDTLADAVGSIPYAPMVVVNLGFAPGTTPVPDGFGFLVPSEEKRALLGAIHTSTTFPFRAPPGH
VLYTCMMGGARQPERASQDVDSLAALAREELRALAGVTATPVLTDVVRWPRGIPQYNVGHLARMAAVDAL
VARWPGLHLTGNAYRGVGLNDCIVHGCELAEQLATH

>**WP_050724738.1**_Vulgatibacter_incomptus_Proteo_delta
MRVAIVGGGISGLALAERLAAAGAEPLVLEADDRAGGKIATRRKDGFLLEAGPNGFLDKEPATLELASRI
GLRDSLRQAETAAKRRWVFVRGALREVPSTPPAFLRSDILPPFAKARVALEPFSRRAKPGVDESIADFGR
RHLGARATRDLLGAMVLGIFGGDVEKLSLASCFPKMAELERAHRSLVLGMIRLQREKKGAGGPAGPGGVL
TSVEGGLGHYPARLAETLGSAVRTGVRVDALSVTESGVRLHTNSGGRAAELDADAVAITAPADVASRLLA
PLDPALGELAAGVPYAPMAVVHLAWPRARIAHPLDGFGFLVPPHEGRGILGAIFVSSIFPWRAPPDQALF
TVMIGGAVRPELAARPEAELATLAASELGGIIGASGDPSLAEVIRWQRAIPQYVIGHEARRREAMERIAR
LGPVHLGGNAWRGIGVNDCIAAAAPLATEILDR

>**WP_169344102.1**_Pyxidicoccus_fallax_Proteo_delta
MKVAVVGGGISGLVIAYRLRSRGRDAVLLESSSRLGGAVGTRARDGYLVEQGPNSFLDREPATRELAAAL
NLEGRIRAADPAAKRRYVYTRGRLRSVPASPPAFLGSDILPLGARLRVMGELFTGRAPEGVDESLAQFGR
RHLGRMATEVLLDAVQTGIYAGDVERLSVAATFPPLVKLEREHRSLILGAIRTQKAQRAQQKALPAGTGV
TGGTAAPPKLSGALSTFEGGLQTLIGALTAALGDAARVGARVEGLERVEGGWRLAVEERGQRSELTAEQV

VLAVPAHVATRLLHPLDATLAARVGEIEYAPIAVVHLGFDAGTTPAPDGFGFLVPSGEKRRLLGSIHAST
TFPFRVEGGRVLYTCMVGGARQPELVKLEEPALAQLAREELKALAGVTATPSFSEVIRWPAGIPQYNVGH
LERVAAIDAALQRWPGLHLTGNAFKGIGLNDCIRNGLQLADTLAGR

>**WP_120605240.1**_Corallococcus_carmarthensis_Proteo_delta
MTVAVIGGGITGLALAHRLRARGTAAVVLESTSRVGGNVQTHARGGYLLEAGPNSFLDREPTTRELAETL
GVSSRIRPADAAAKNRYVFTRGALRALPASPPAFLKSDILPLAARLRVLGELFSGRNPTGSDESLAQLGR
RHLGREATSVLLDAMQTGTYAGDPEQLSAEATFPQLVKFEREHRSLILGAIRAQRAARQAKASGTAEAGP
ALTGQMSTFDGGLGVLVDALAKALGGAVRTDAKVEGLERTADGWKVRFREQGQPAELTASHVVLAVPAHV
AAELLRPLDAELAQKADSIPYAPIAVVHLGFAAGTVPKPDGFGFLVPAVEGKALLGTIHVSTTFPFRVEG
GRVLLTCLMGGARRPEVVARDEDALAALAREELKTMAGLTATPELTEVIRWPRGIPQYTVGHLERLAAME
ERLKRWPGLHLTGNAYRGVGVNDCIREATRLADALGGQAEGAVRSA

>**WP_002619304.1**_Stigmatella_aurantiaca_Proteo_delta
MAVIAVVGGGITGLALAHRLRSRGKDAVVLEAGAHLGGVIQTRQRDGFSTETGPNSFLDREPATRELAAS
LGIEERIRMADPSAKSRSLYTRGQLRPVPASPPAFLKSDLLPLGTRLRVLAELFTGRAPPGQDESLGDFG
RRHVGARATSVLLDAMQTGTYAGDVEALSAEAAFPTLKQLEREHRSLLLGAVRTQGRQRAPAPAGTKLKG
AMCTFEGGLGTLVEALARALGPAARTGAAVEGLARSQNGWRLSVRERGQQAELEASQVVLTSPAHVSAEL
LAPLDPSLAGHLKGIPYAPIAVVHLGFAPGKTPPPDGFGFLVPGQEQRQLLGVIHVSTVFPFRAEGGRVL
YTCLMGGARRPDLVGLNEEALAALAQQELREMAGVTASPDFTEAVRWPRGIPQYTVGHLERLSAIDSALA
RLPGLHLAGNAYKGVGLNDCIRNAAALAETLASR

>**WP_002629683.1**_Cystobacter_fuscus_Proteo_delta
MRVIIVGGGISGLVLAQGLRARGTEVTLLEAGAQPGGNIQTHRRDGFVTEAGPNSFLDREPSLRALAARL
GIEDRIRTADPSAKRRYLYSGGKLRALPQSPPALLKSDLLPWTAKLRMLGEPLTRRGPTGDESLADFGRR
HVGSAATEVLVDAMQTGIYAGDMEALSVGAVFPKVAQLEKQHRSLLLGMVRERKAERAAPPPAGTPATTG
AVASFDGGLGVLVKALAGALGPALRLEARVVGLRREAAGWRVAVEERGQRTELEADRVVLAVPAFTAAEL
LRPLDATLATQLDGITYAPIAVVHLGFAPGAVPPPDGFGFLVPAVEKRRVLGVIHVSSTFPWRTEGGRVL
YTCLIGGARRPDLVELDEAALVTLAREELRLMAGVTAEPVLTETIRWKRGIPQYNLGHLGRLAAIDEGVT

RLPGLFLTGNAYRGVGLTDCVREATGLVDALAR

>**CAH1247353.1**_Branchiostoma_lanceolatum_Eukaryota

MTSVAVIGGGISGLSAAYYLSKAPHLVNKVVLLEGSSRLGGWLQSTRTEEGAIFEHGPRSLRVAGEPGAN

ILEMADDLGLSDEIVPVLPSHEGAKNRFIFAGGKLHKLPSSFRGLFSRYELFGNQSPALLGLREPFIKRR

EEETDESVHSFFCRRLGKQFTENAIDPMVRGIYGGDCRQLSVQALFPSMHQAEKRKGSITRGLLFGPKQK

ETPFRIDSELLKKAKKEQWALWSLKDGLEGLSDSLKGHLARSGVELLTERRVERLEFDTTNQVVQVNTLD

EQFQVNHVISAVPSNCLSPMFAGHHPVLSDNLSANPSATIGLVNLEYAGDVLPSEGFGYLVPSGEPERIL

GVVFDSSIFPQHNRPSSATTRLTVMMGGTWFNQLFGDPDKVDSSLLLDVAVTTVGQHLKITSEPLRSFTT

IQKDCIPQYTLGHTDRLEQMESYIAERSLPLSLVGCSYRGVGVNDCVLSARKAVTDFLKTCERD

>**AAI54349.1**_Danio_rerio_Eukaryota

MQKVVAVLGGGIGGLSACHHLSKSPNVSKIVLLEGSGRCGGWLSSVRRDDGAVFEQGPRGVRPGGAVGRN

TLNMVSELGLESELLPITSDHLASQNRFLYVKGQLHKMPSGLGGVLRTIPPFSRPIIQSVLKELVISKGT

EEDESVHAFVSRRLGSELADIAIDCLCRGVFAGDSRQLSVRSCFPPLYEAEQARGSIVLGMLMGSGAGPK

VVPSGLAKRASKESWTQWSLKRGMQTLPEALEDSLRRRNGVELHHHAKVKRLNVDSTGWEIKLDDGTSLK

ADHVISTLPASALASVLPPAAQTLSEQLRSIASVNVALVNLEYEGFILPVTGFGHLVPSTEDAGVLGVVY

DSVPFPEHNRSGGATTRLTVMMGGAWFEQTFGSPDLVMEQTLLDRAVQAVNSHLSVTSQPVWSCVALLKN

CIPQYHLGHWKRLEKMRQYISNHNLALTLAGASYDGVSVNDVIFSGRTAAEGLVGKI

>**NP_001167359.1**_Salmo_salar_Eukaryota

MQKTVAVLGGGIGGLSACFHLSKSPQVSKIVLLEGDGRFGGWLSSTRREDGAVFEHGPRGIRPAGAVGRN

TLNMVEELGLESEVLPVTYDHVASKNRYLYVGGQLHKMPSGLGGVVRTVPPFSRPLIQSVLKEILISRGK

EEDESVHSFVSRRLGTELADIAIDSLCRGVFAGDCRKLSVRSCLPPLYNAEKARGSIVLGMLLGSGPGPD

VPPSTLAKRAAQENWAQWSLKRGLQNLPEALEERMRNGGCVEVHRDTPVTGLSTNGTGWEIQLEDGTIKA

DHIISALPAKALASALPSAAQPLSQQLLEIATVTVAVVNLEYEGSVLPVTGFGHLVPSSEDRGLLGVVYD

SVPFPQHNRTVGPTTRLTVMMGGAWFQEVFGNPDEVTEQLLLDRATQAVTSHLGVTTPPIWSIVALLKDC

IPQYYLGHWKRLENMRQYIRDHNLPLSLAGSSYDGVSVNDVIFSGRTAAEGLVGKV

>**XP_050845225.1**_Vespula_vulgaris_Eukaryota

MTVVLGGGISGLSAAYYALNNPRIGPIIVLEASNRLGGWIRSHELSNGIIFEKGPRTIRCLGLAGKNTLN

LLEDLQLTNKLIPIKSDHPSAKNRMIYSRNKLHLLPNSFLSIIKTNSLLNRSLASFLVKDLIAPMVRKDD

ESIYSFTTRRIGKDIADYLISPMICGICAGDAQKISVNFLMKSLFEAEQKHGSITKGLLKQLFKKKNVNN

KIEYKSNLAKRADTEKWVVWGLKGGLEELPRSLGNNLQSQGVDIQLNSHCEKLFFQSNHVELIINGKARK

CSHVISSLPAKTLAKLLEEQHPDLAEQLRSIPMVTVAVVNLQFADNVLPMNAFGFLVPPGENLPILGVIF

DSCIFPKDSSTVLTVMMGGAWFEKYFGKDPSDENLLNIAIKQVKEILNIKKEPIAFNVAILKDCIPQHVV

GHAQRLNHIRDYISSRRIPLALCGSSYQGVGINDVILSAKEAVSEIVQHSNELKN

>**XP_026546008.1**_Notechis_scutatus_Eukaryota

MQLKVAVLGGGVTGLAACYYLARSSLASKVTLLEGSHRLGGWIHSTRTEDGAVFEHGPRGIRPAGLVGKN

TLLMISELGLEADVLPVPGDHPASKNRYLYVGGSLHKLPSGLKGIFQTVPPFSSPLVWSGLKELWAPRGT

EPDETIHAFVARRFGQEMADIAIDSLCRGVFAGDSRALSIRSCFPALFLAERKHGSVMLGMTLAKKETSP

VDCRLIRQAREGHWSQWSLRGGLETLPQSLASFSRERGVKIHCNALVKRLDRTTSGSWQIALQDGNMEAD

HVISALPARVLADLLPARFEPLIQDLLAIQAVSVAVVNLQYENAQLPVTGFGHLVPSFEDRPLLGIVYDS

VAFPEQNGHQGSATRLTVMLGGAWFTSHLGDPNTIPHSELLSRAQEAVRKHLGISAEPVRSIVKVHQSCI

PQYTLGHWKHIESATSQLKQHNLPLSLVGASYDGVSVNDCIFSAQTAVSRLVGGVS

>**WP_230840699.1**_Gloeobacter_morelensis_Cyanobacteriota

MSTAEELLDVLVVGAGLSGLALAWNLKRAGCTLLICQAGERVGGAITTALTDGFVCEGGPSSFQESPALI

ELLTQLQLEDQIVTADPRLARFVWWENRLRSVPLTPPQLVRSDLLSWSGKARLLWELFVPALGEPREETV

AEFVLRRFGEEVLSRLVDPLVSGMCAGDVGQLSVEATFEGLVELERRHGGVLRGLWRTARTRAPLKRLCT

LRGGLEQLPQALAQRLQPQLLLSHRLEALERLSGDHWRAVVAGPQGEPLAIAARTVILAGAAHAMAPVLR

PLDAGLGRALESIYYPPVASINLGYSKSQVPNAPEGFGHLIPRNQTLRSLGVIWNSSLFPHTAPPNWRLY

TCFVGGTTDPATPNLSDTELASLAHRELQTALGFQAGYQLLRVTRWPQAIPQYALGHPSKQERVERALLG

LPGLFLAGNYFGGISLGDCVRHSGAVASRVLQFLSTVASNGSLRPA

>**WP_017328280.1**_Synechococcus_sp_PCC_7336_Cyanobacteriota

MGSHTPDILILGAGISGLSAAFRLHQQQQDLLVAERAERVGGVITTRAQDGFRWEEGPNSFTPSPALLNL

IADAGIADRLLWADGKLPRFVYLEGKLTLVPMTPPDLIKSNLLSFGAKLRALLGILGFTAKAPDKEETVE
EFFARQLGPQVVERLVGPFTSGVYAGDTQQLSATAAFSKVADLERKYGSIIAGIIRSPKSPKPPISAKID
PLPKRGQLGNFVEGLQELPDAIAQQLGDAVKLQWEAAEIVKEGDRYRTTFQTPSGPQTVSSKAILLAVPA
YRAAPLLKSLDTALADELAAIPYPHVGAVTLAYPADALPQPFAGFGQLFPRGQGIRTLGTIWTSSLFPGR
APAGYQCTLSYIGGATDPDIAQMTDEALARTVHQDLSKTLLVKEAEPRVMGVRRWPRAIPQYTLGHRQRL
ARIDELLADYSGLVLCTNYLDGVALGDCVRRGEARAADLVEWLAQAE

>**WP_249101636.1**_Argonema_galeatum_Cyanobacteriota
MLDTLIVGAGISGLSLAHALQQDGRRILLCERQGRVGGNITTGMAEGFLWEEGPTSFSPTPELLRLAVDV
GLEQELMLADRKLPRYVYWQGQLQPVPMSPPAAISSQLLTLQGKLRALTGALGFIAPAMALSEQGGEETI
AQFFQRHLGAEVTQRLVAPFVSGVYAGDPDLLSASASFRRIVKLSEMGGGLLAGAILQRRGSKKQGKPQD
PSIPKTRPGELGSFKQGISALPQAIASHLGDTVKLGWHLTTLQKSDRQTYIAEFDTPAGPEQVEACTIVL
TTPAYVTADLLQFLIPTASQVLREIPYPPVACVVVAYPQTALKQPLRGFGNLIPRRQSIRTLGTIWSSSL
FPGRAPQGWQLLTNFIGGATDPEIADLDDEQIVQAVHQDLYRILLKEDVAPKVLAVHLWKRAIPQYTLGH
LQRLEQVNQALEQFPGLFLCSNYFDGVSLGDCVKRAIELSAEIRQHLQ

>**WP_146295316.1**_Euhalothece_natronophila_Cyanobacteriota
MLDSLIIGAGISGLSTAYRLQEKNQEILVTEKRDRAGGNIISQQQGEFLWEEGPNSFSPTPELLKLAVDV
GLKDEFVFADRALPRYIYWQGKLRQVPMTPPAAITTPLLSPLGKLRALSGALGFAPPKVTNQPETVAEFF
SRNLGSEVAERLVSPFVSGVYAGDVNQLEAAAAFGRVTKLADTGGGLVAGAILSRGKKKKPAATDNKEIP
ETKSGELGTFKQGLQQLPSAIASKLGDNLKLNWELQSLSRHPEQGYIAEFSTPEGQQTIEAKTVVLTTPA
HVASPLLQDISPESSNALAEIPYPPVACVVLAYPIDALRFPLNGFGNLNPRSQNLRTLGTIWSSILFPGR
TPQGWYLLTNFIGGATDPEIAQLSEEEIIQQVHQDLEKVLVKPNTEPKPLAVRLWSKAIPQYNVGHLERL
KTAKDGLKDLPGLFLCSNYLDGVALGDCVRRGEETSQAILEITKATSGA

>**WP_017303635.1**_Spirulina_subsalsa_Cyanobacteriota
MLDSLIVGAGISGLSAAHTLQKQQTQFLVTESQGRVGGNITTNRQGDYLWEEGPNSFAPTEDLLRLAVEV
GLKEDLVFADRRLPRFVYWNQQLHPVPMSPPAALKTQLLSEAGKWRAALGALGFVGGLVGREEETVRQFF
TRHLGTEVTERLVAPFVSGVYAGDVDQLSAQAAFRRVFEFAQLGGGLAAGGILARRQAPPKAPPDPSLPE

TKTGQLGSFREGLEMLPRAIASQLGDRLKLQWRLTHLEITPQQTYLAHFNTPDGPQQIATRTLILTTPAP

ITADLLKPLTPALHGVLKEIYYPPVACVVLAYPRAASARPLEGFGHLIPRNQGIRTLGTIWSSCLFPGRT

PEGEHLLTNFIGGATDPGIAQLDPEEIAQAVHQDLCKILIRPEFTPKILAVRLWKQAIPQYTLGHLQRLA

TLEQELSKFPGLHILANYTDGVALGDCVKRGVAVAQKIRSRE

>**WP_009787090.1**_Lyngbya_sp_PCC_8106_Cyanobacteriota

MTHVLDSLIVGAGISGLALAHALHQNQDHQLPLNILVSEHQGRVGGNITTVSEGEFLWEEGPNSFSPTPE

LLKLAVEVGLKPELVFADRKLPRYVYWNGQLMPVPMSPPALLSTKLLSPGGKLRALTGALGFVQPAMGES

LSQQNGEETISQFFERHLGSEVLKRLVEPFVSGVYAGDPQQLEISSAFARVARMAYSGGGLVAGAVLSRR

QNKSPRSPADPSIPQTKRGELGSFRQGIGALPNAIAKQLGDQLKLNWQLTRLERTENQTYRAEFSTPEGV

QQVETRTVVLTTPAYVTAEILKPLQLQVSQTLTEIPYPPVACVVLAYPVSALKQKLTGFGNLVPRGQGIR

TLGTIWTSSLFPGRAPQGWQVLTSYIGGATDPEIGELEDDQIVEAVHQDLRHILLKEDISPKVLAVHLWK

RAIPQYNLGHQQRLQHVNEGLEAMPGLYLCSNYIDGVALGDCVRRSIGQANEILSFLGQ
